# Comparing the efficacy of physical therapy interventions in Alzheimer’s disease: a network meta-analysis

**DOI:** 10.3389/fnagi.2025.1541287

**Published:** 2025-03-05

**Authors:** Jiawen Wu, Yunfei Teng, Yaming Xie, Shuangtao Xing, Songsong Zhi

**Affiliations:** ^1^School of Music and Dance, Henan Normal University, Xinxiang, China; ^2^School of Physical Education, Henan Normal University, Xinxiang, China; ^3^School of Environment, Henan Normal University, Xinxiang, China

**Keywords:** Alzheimer’s disease patients, mesh meta-analysis, physical intervention, music therapy, intervention effect

## Abstract

Alzheimer’s disease (AD) is a progressive and debilitating neurodegenerative disorder that significantly impairs cognitive function and daily living abilities, representing a major public health challenge. Given the multifactorial nature of AD, effective therapeutic interventions targeting both cognitive and functional decline are critical. This study aimed to conduct a comprehensive comparison of the therapeutic effects of music therapy, acupuncture therapy, game therapy, cognitive training therapy, and exercise therapy on AD patients through a network meta-analysis. Randomized controlled trials (RCTs) published up until 2024 were systematically retrieved from multiple databases. Data were extracted, including the first author, publication year, country, total sample size, mean participant age, type and duration of intervention, and outcome measures such as the Mini-Mental State Examination, Activities of Daily Living, and Alzheimer’s Disease Assessment Scale-Cognitive Subscale. Statistical analyses were performed using the RevMan 5.3 and Stata 17 software. The analysis included 52 RCTs with a total of 3,409 participants, offering a strong dataset. The results indicated that game therapy produced statistically significant improvements in mental state and daily living abilities, while acupuncture therapy yielded the most pronounced improvements in cognitive function among AD patients. Notably, the comparative efficacy of these interventions suggests that game therapy may offer short-term benefits, particularly for mental health and functional abilities, whereas acupuncture therapy demonstrated superior long-term cognitive enhancements. In conclusion, tailored physical and cognitive interventions such as game therapy and acupuncture therapy may hold significant potential in optimizing treatment outcomes for AD patients, with implications for both clinical practice and future research.

## 1 Introduction

Alzheimer’s disease (AD), a progressive and irreversible neurodegenerative disorder, poses a serious threat to global public health and is the fourth leading cause of death among the elderly (Villain and Michalon, 2024; Chen et al., 2021; Hall et al., 2024). The worldwide prevalence of AD is rapidly escalating due to aging populations. According to the most recent statistics from the World Health Organization (WHO), the number of people living with AD was 50 million in 2018, (Joseph et al., 2021) and this number is expected to rise to 131.5 million by 2050 (Prince et al., 2015). This increase will place substantial pressure on healthcare resources, requiring urgent attention from clinicians and policymakers. Pathologically, AD is characterized by extracellular accumulation of amyloid beta-protein (Aβ) forming senile plaques, abnormal phosphorylation of tau protein, and neuronal loss. The “Aβ cascade hypothesis” posits that the abnormal accumulation of Aβ in the extracellular space is central to the pathogenesis of AD. The increased Aβ leads to chronic neuroinflammatory responses, synaptic dysfunction, oxidative stress, and neurofibrillary tangles, all contributing to neuronal structural and functional abnormalities (Sancheti et al., 2013; Chen and Zhong, 2013). In AD, the neurons affected by neurofibrillary tangles are primarily found in specific regions of the brain, including pyramidal neurons in the CA1 area of the hippocampus, as well as neurons in the medial prefrontal cortex and temporal cortex (Zhang and McGeer, 2004). These pathological processes result in deficits in cognitive domains such as memory, executive function, language, and visuospatial abilities, as well as impairments in physiological function. As the disease advances, patients not only experience cognitive decline but also face an increased risk of psychiatric conditions such as depression, anxiety, and behavioral disturbances, further complicating their care (Fang et al., 2006).

Currently, pharmacological interventions for AD are primarily aimed at slowing the progression of the disease, as no available treatments can halt or reverse its development (Zhang, 2019). The most commonly prescribed drugs, such as cholinesterase inhibitors and NMDA receptor antagonists, offer only limited symptomatic relief and are associated with a range of adverse effects, including gastrointestinal disturbances, dizziness, and cardiovascular complications. Furthermore, the long-term efficacy of these drugs remains uncertain, with some patients showing minimal response (Connors et al., 2018; Hessmann et al., 2018). Suboptimal medication adherence is another significant challenge, as the burden of managing chronic pharmacotherapy often leads to poor compliance, which exacerbates psychological stress on patients and caregivers and limits the effectiveness of treatment (Wang et al., 2022).

In recent years, the limitations of pharmacological interventions have prompted increasing interest in non-pharmacological therapies. As a non-invasive treatment modality, physical therapy avoids the risks associated with surgical procedures and the adverse effects of long-term medication use. Physical therapy interventions have demonstrated promise in improving both physical and cognitive outcomes in AD patients by promoting neuroplasticity, enhancing cerebral perfusion, and stimulating neurogenesis (Shufen and Hongxia, 2011). These therapies encompass a wide range of approaches, including music therapy, acupuncture, game therapy, cognitive training, and exercise therapy. For instance, exercise therapy, particularly aerobic exercise, has been shown to slow cognitive decline and improve cardiovascular health, (Xu Q. et al., 2019) while cognitive training helps maintain executive function and attention by stimulating neural networks (Luo and Xu, 2017). Similarly, music therapy has been associated with improvements in mood, emotional regulation, and social interaction, providing holistic benefits that extend beyond cognitive domains (Liu et al., 2019).

Moreover, the mechanisms underlying these physical therapies suggest that they may induce long-term neuroprotective effects. Exercise, for example, increases levels of brain-derived neurotrophic factor (BDNF), which supports the survival of existing neurons and the growth of new neurons and synapses in the hippocampus, a critical region for memory (Gu and Wang, 2019). Acupuncture has been proposed to modulate neurotransmitter systems and reduce the accumulation of amyloid-beta plaques, a hallmark of AD pathology (Yang et al., 2023). Given these potential benefits, physical therapy represents a promising adjunctive treatment option for managing AD.

Existing meta-analyses have predominantly compared the effects of individual or combined interventions, often using routine care or no intervention as the control group. However, these traditional meta-analyses have limitations in that they typically only allow for pairwise comparisons between two interventions. This can leave significant gaps in our understanding of the relative efficacy of multiple treatments, particularly in complex conditions like AD where multiple therapeutic approaches may target different aspects of the disease. Network meta-analysis (NMA), on the other hand, is a more sophisticated technique that allows for the comparison of several interventions simultaneously, integrating both direct and indirect evidence to provide a more comprehensive evaluation and ranking of treatment options (Owen et al., 2020). This method enables the identification of the most effective interventions based on a statistical approach that synthesizes data from various studies.

Consequently, this study undertakes a network meta-analysis encompassing five distinct types of physical interventions—music therapy, acupuncture, game therapy, cognitive training, and exercise therapy. By employing the Surface Under the Cumulative Ranking (SUCRA) approach, the interventions are ranked according to their efficacy in improving cognitive and functional outcomes in AD patients. The findings are expected to provide valuable evidence-based guidance for clinicians in selecting the most appropriate treatment strategies, while also offering insights for future research to optimize non-pharmacological therapies for AD.

## 2 Materials and methods

### 2.1 Search strategy

A comprehensive and systematic literature search was conducted to identify relevant publications across multiple databases, including China National Knowledge Infrastructure (CNKI), Superstar, VIP, Wanfang, PubMed, Cochrane Library, and Web of Science. The search covered all available publications from the inception of these databases until 2024. The search strategy was carefully designed to ensure inclusivity, utilizing both Medical Subject Headings (MeSH) terms and free-text keywords, to capture a wide range of studies relevant to physical therapy interventions in Alzheimer’s disease (AD). The key search terms were: (Music therapy OR acupuncture therapy OR play therapy OR cognitive training therapy OR exercise therapy) AND (Alzheimer’s disease OR dementia) AND (cognitive OR mood OR depression OR anxiety OR quality of life OR lifestyle). Additionally, backward and forward citation searches were employed to identify further relevant studies. Articles that met the inclusion criteria were obtained via direct download or interlibrary loan services.

### 2.2 Inclusion and exclusion criteria

The study design was limited to randomized controlled trials (RCTs). (1) Study population included patients diagnosed with Alzheimer’s disease, senile dementia, or cognitive impairment, irrespective of gender or nationality, who were conscious and free from severe cognitive deficits. (2) The experimental interventions comprised music therapy, acupuncture therapy, play therapy, cognitive therapy, and exercise therapy, while the control group received standard treatment, routine care, or daily activities. Detailed definitions of the physical interventions and control groups are provided in Table 1.

**Table 1:** Definitions of physical therapy and control groups.

Intervention measure	Definition	Details
Physical therapy	Music Therapy	Incorporates music as a therapeutic tool to promote health and facilitate recovery.
	Acupuncture Therapy	Utilizes traditional Chinese medicine techniques, specifically acupuncture, to balance the body’s vital energy (qi) and improve blood circulation.
	Play Therapy	Engages play-based interventions to elicit adaptive responses within neuromuscular systems.
	Cognitive Training Therapy	Psychotherapy focuses on altering negative cognitive patterns through cognitive and behavioral techniques.
	Exercise Therapy	Employs physical exercise as a modality for disease management.
Control groups	Standard Therapy	Includes pharmacological treatment, standard nursing interventions, daily activities, or non-physical interventions

Inclusion criteria were as follows: (1) The study design must be a randomized controlled trial. (2) Participants must meet the diagnostic criteria for Alzheimer’s disease as defined by the American Psychiatric Association for the Elderly (Connors et al., 2018). (3) The intervention must involve one or more of the following therapies: music therapy, acupuncture therapy, play therapy, cognitive therapy, or exercise therapy. The control group should receive standard treatment, standard nursing care, daily activities, or non-physical interventions. (4) Outcome indicators must include; ① Cognitive function: Mini-Mental State Examination (MMSE); ② Alzheimer’s Disease Assessment Scale-Cognitive Subscale (ADAS-cog); ③ Activities of Daily Living Scale (ADL).

Exclusion criteria were as follows: (1) Non-randomized controlled trials. (2) Conference proceedings, master’s theses, doctoral dissertations, abstracts, and duplicate publications. (3) Studies with missing or unreported required data (e.g., means, standard deviations, etc.). (4) Inaccessibility of the full text. (5) Studies with low-quality physical interventions or lacking rigorous experimental design. (6) Studies involving subjects not diagnosed with Alzheimer’s disease or AD-type dementia. (7) Interventions that combine multiple physical therapies (e.g., exercise combined with cognitive training, music combined with exercise) versus routine nursing as a control. (8) Animal experiments. To ensure robust and high-quality data, studies were screened for methodological rigor, including appropriate randomization, blinding, and adherence to intervention protocols.

### 2.3 Literature screening and data extraction

Two researchers independently screened and selected relevant literature in a double-blind manner. Data from the selected studies were extracted by both researchers, followed by mutual verification to ensure accuracy. The extracted data were then standardized using SPSS software, and any unstandardized data were processed accordingly before the mutual verification. In case of discrepancies, a third researcher would resolve the conflict through discussion and review. The extracted data included the first author, publication year, country of origin, total sample size, participant age, exercise intervention details, duration of the intervention, and outcome measures. Specifically, data extraction included recording the means, standard deviations, and sample sizes for both the experimental and control groups post-intervention.

### 2.4 Quality assessment of literature

Two researchers independently used the ROB 2.0 Risk of Bias Assessment Tool to assess the quality of the included studies (Hu et al., 2024). The assessment covered seven domains of bias: (1) random sequence generation, (2) allocation concealment, (3) blinding of participants, (4) blinding of outcome assessors, (5) completeness of outcome data, (6) selective reporting of findings, and (7) other potential sources of bias. Each domain was rated as “low risk,” “high risk,” or “unclear risk.” The evaluations were then cross-verified by the two researchers. In case of disagreements, a third researcher mediated to reach a consensus on the study’s inclusion.

### 2.5 Statistical methods

This study used Stata 17 and RevMan 5.3 software to conduct a network meta-analysis of the selected data indicators. The study strictly adhered to the guidelines set forth in the Preferred Reporting Items for Systematic Reviews and Meta-Analyses (PRISMA 2020). The effect size for continuous variables was expressed as the standardized mean difference (SMD) with the corresponding 95% confidence intervals (CIs). The heterogeneity between independent studies was assessed based on the I^2^ statistic and the significance level (*p*-value). Specifically, I^2^ < 50% and *p* > 0.1 indicated no significant heterogeneity, allowing the application of a fixed-effects model; conversely, I^2^ ≥ 50% and *p* < 0.1 indicated significant heterogeneity, necessitating sensitivity analysis. To address the issue of inconsistent data units, unit conversion was carried out using the calculator feature in RevMan 5.4. The Surface Under the Cumulative Ranking (SUCRA) values were evaluated using cumulative probability plots to facilitate the ranking and comparison of various exercise interventions, with a range of 0 ≤ SUCRA ≤100. A SUCRA value of 100 indicates the best effect, while 0 indicates the worst or no effect, with higher SUCRA values suggesting better intervention outcomes. SUCRA values for mental status, daily living abilities, and cognitive assessments were systematically evaluated across different physical therapies. Cluster stratification analysis was used to identify the most effective exercise intervention, ensuring a comprehensive evaluation and comparison of the interventions. This systematic approach enabled the identification of the most effective exercise strategies for improving outcomes in Alzheimer’s disease patients.

## 3 Results

### 3.1 Literature screening process and results

**Figure 1:**
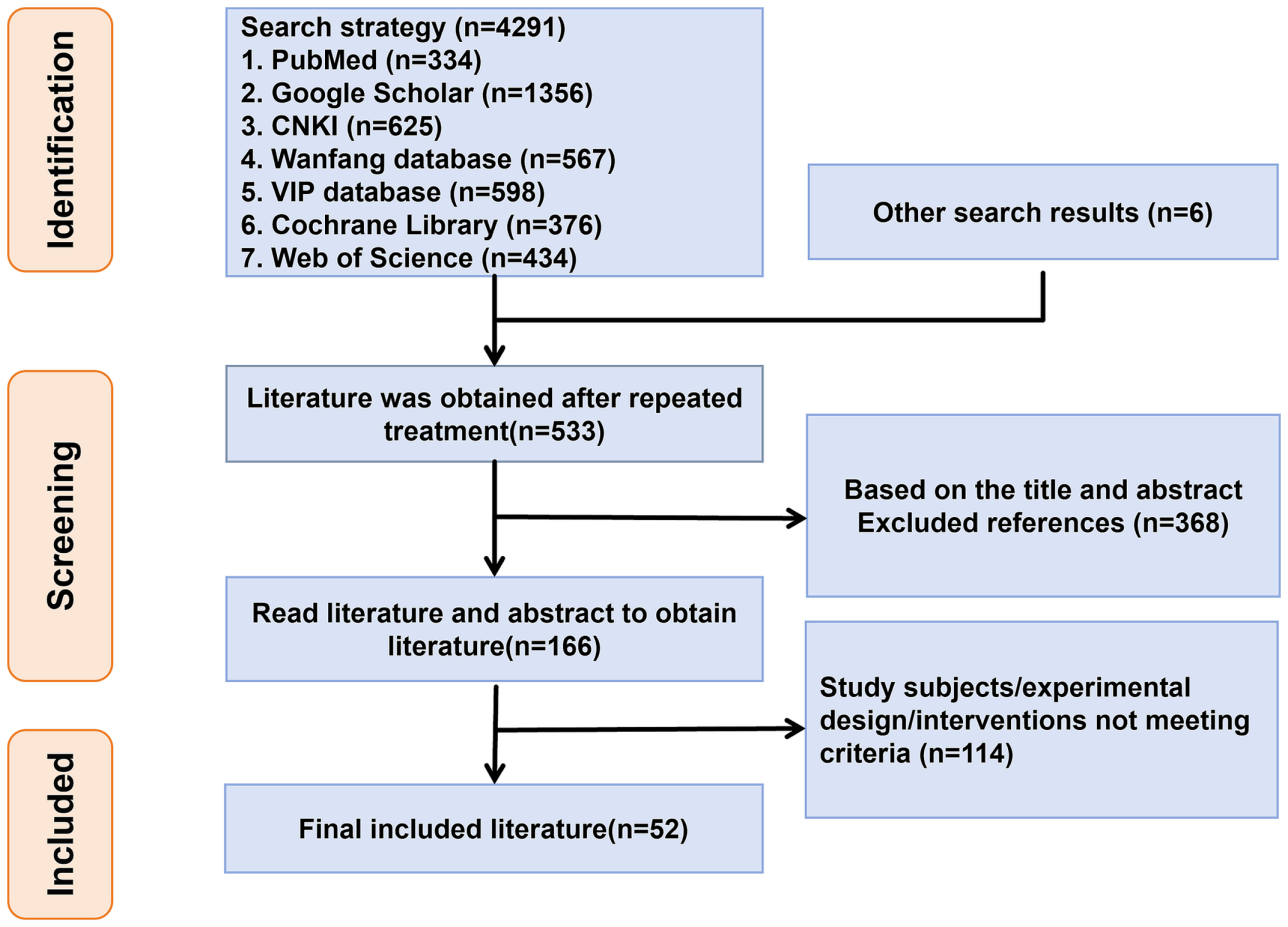
Flow chart of literature search and article inclusion.

A comprehensive search across multiple databases, including PubMed (*n* = 334), Google Scholar (*n* = 1,356), China National Knowledge Infrastructure (*n* = 625), Wanfang (*n* = 567), Weipu (*n* = 598), Cochrane Library (*n* = 376), and Web of Science (*n* = 435), initially identified 4,291 articles. After removing 978 duplicates, 3,313 articles remained for title and abstract screening. A rigorous multi-stage screening process was implemented, starting with the exclusion of studies that did not meet the inclusion criteria (e.g., non-randomized studies, unrelated interventions, non-AD populations). Ultimately, 187 full-text articles were reviewed for eligibility. Following detailed evaluation, 52 RCTs comprising a total of 3,409 participants were included in the final analysis. This selection process is visually summarized in Figure 1, and the key characteristics of the included studies, such as sample size, intervention type, duration, and outcome measures, are provided in Table 2. This comprehensive screening ensures a robust evidence base for conducting a network meta-analysis and limits the risk of selection bias.

**Table 2:** Main study characteristics.

First author and year of publication	State	Sample size	Age (years)		Intervening measure		Intervention cycle	Outcome indicator
		T/C	T	C	T	C		
Wang et al. (2022)	China	35/35	72.13 ± 2.92	71.52 ± 2.83	I	VI	8 week	①②
Yuan (2018)	China	23/23	67.90 ± 8.10	68.20 ± 7.80	I	VI	8 week	①
Xiao et al. (2018)	China	25/25	73.89 ± 7.52	72.37 ± 7.73	I	VI	16 week	①
Qu et al. (2018)	China	41/41	79.42 ± 13.44	78.58 ± 12.14	I	VI	12 week	①②
Liu and Liu (2017)	China	12/13	60.78 ± 3.40	70.13 ± 4.10	I	VI	10 week	①
Zhong et al. (2015)	China	28/29	78.10 ± 7.70	78.15 ± 6.50	I	VI	24 week	①②
Meng et al. (2019)	China	21/21	67.80 ± 2.60	66.40 ± 3.90	I	VI	8 week	①
Chen et al. (2020)	China	21/22	83.00 ± 5.03	85.18 ± 5.59	I	VI	12 week	①②
Innes et al. (2021)	America	20/20	66.85 ± 2.14	61.45 ± 1.38	I	VI	12 week	②
Gomez-gallego et al. (2021)	Spain	28/41	83.93 ± 8.01	78.67 ± 5.73	I	VI	12 week	①②
Cheour et al. (2022)	Spain	7/6	76.23 ± 4.27	76.23 ± 4.27	I	VI	16 week	①
Flo et al. (2022)	Norway	21/14	65.00 ± 9.80	69.80 ± 10.60	I	VI	40 week	①
Jung et al. (2020)	Korea	59/59	77.20 ± 7.10	77.20 ± 7.10	I	VI	8 week	①②
Du et al. (2019)	China	41/39	66.23 ± 9.65	67.04 ± 8.68	II	VI	3 week	①
Zhang X. et al. (2019)	China	40/40	62.00–91.00	62.00–91.00	II	VI	12 week	①②
Yang (2019)	China	44/44	71.63 ± 4.82	71.89 ± 4.72	II	VI	12 week	①②
Zheng and Chen (2018)	China	18/20	81.74 ± 5.79	84.26 ± 5.48	II	VI	8 week	①②
Zhu et al. (2020)	China	35/41	72.78 + 3.02	73.05 ± 3.11	II	VI	8 week	②
Chen et al. (2016)	China	68/75	82.57 ± 5.37	82.57 ± 5.37	II	VI	24 week	①②
Li and Li (2017)	China	19/21	83.10 + 4.10	81.80 + 6.70	II	VI	16 week	①②
Padala et al. (2012)	America	15/15	72.10 ± 5.30	73.90 ± 7.10	II	VI	8 week	①②
Santen et al. (2020)	Netherlands	73/39	79.00 + 6.00	79.00 + 7.00	II	VI	24 week	①
Yang and Kwak (2017)	Korea	10/10	71.10 ± 6.90	69.90 ± 8.70	II	VI	12 week	①
Xu L. F. et al. (2019)	China	40/40	67.78 ± 5.00	68.12 ± 4.41	III	VI	3 week	①②
Wang et al. (2021)	China	30/30	69.40 ± 4.83	68.47 ± 5.10	III	VI	8 week	①②
Feng et al. (2019)	China	20/20	69.00 ± 7.00	68.00 ± 9.00	III	VI	12 week	①
Chen et al. (2017)	China	51/51	68.59 ± 4.36	68.59 ± 4.36	III	VI	16 week	①②③
Jia et al. (2017)	China	35/36	74.50 ± 6.83	75.11 ± 6.53	III	VI	12 week	①②③
Bi et al. (2016)	China	37/37	70.20 ± 4.60	71.50 ± 4.70	III	VI	12 week	①
Liang (2016)	China	37/37	73.80 ± 6.70	72.50 ± 5.20	III	VI	12 week	②
Xia et al. (2020)	China	30/30	61.00 ± 8.00	62.00 ± 7.00	III	VI	8 week	③
Sun et al. (2018)	China	55/55	66.23 ± 4.12	66.31 ± 4.25	III	VI	6 week	①
Guan (2017)	China	30/30	70.50 ± 9.30	69.30 ± 10.20	III	VI	8 week	①②③
Li et al. (2019)	China	29/32	77.34 ± 7.90	77.06 ± 7.04	IV	VI	6 week	①
Wang (2021)	China	58/32	71.72 ± 5.64	72.19 ± 6.82	IV	VI	12 week	①②③
Xu et al. (2020)	China	30/30	66.80 ± 2.89	66.53 ± 2.94	IV	VI	8 week	①②
Hu et al. (2019)	China	37/37	72.34 ± 6.61	71.78 ± 6.45	IV	VI	12 week	②③
Zeng et al. (2019)	China	30/30	54.43 ± 10.72	57.37 ± 10.23	IV	VI	8 week	①②
Zhang et al. (2018)	China	29/29	50.00–80.00	50.00–80.00	IV	VI	24 week	①
Luo and Xu (2017)	China	45/45	70.46 ± 2.38	70.55 ± 2.34	IV	VI	8 week	①②
Wei et al. (2018)	China	48/50	73.09 ± 7.18	72.39 ± 6.89	V	VI	4 week	①②
Wang et al. (2019)	China	18/18	68.70 ± 9.40	71.10 ± 7.70	V	VI	12 week	①
Liu et al. (2017)	China	24/24	70.90 ± 9.20	70.30 ± 7.70	V	VI	12 week	①③
Yan et al. (2015)	China	36/18	/	70.60 ± 7.30	V	VI	24 week	①②③
Mu et al. (2016)	China	39/39	72.90 ± 5.40	73.70 ± 4.60	V	VI	16 week	②
Wu and Huang (2015)	China	46/46	74.00 ± 4.32	73.91 ± 3.99	V	VI	24 week	②③
Chang et al. (2015)	China	27/30	70.74 ± 7.42	70.23 ± 8.52	V	VI	16 week	①②
Qi and Zhang (2015)	China	8/7	71.80 ± 9.10	76.40 ± 5.10	V	VI	12 week	①
Silva et al. (2019)	Brazil	12/7	81.20 ± 8.90	77.50 ± 8.10	V	VI	12 week	①
Fonte et al. (2019)	Italy	20/21	79.00 ± 9.00	80.00 ± 7.00	V	VI	24 week	③
Hoffmann et al. (2016)	Denmark	102/88	69.80 ± 7.40	71.30 ± 7.30	V	VI	16 week	①③
Nie et al. (2019)	China	30/30	73.63 ± 3.96	74.20 ± 3.74	V	I	8 week	①

T/C stands for Treatment/Control. The experimental groups consisted of the following interventions: Music Therapy (Group I), Play Therapy (Group II), Acupuncture Therapy (Group III), Cognitive Therapy (Group IV), and Exercise Therapy (Group V). The control group received routine treatment, routine nursing, or engaged in daily activities (Group VI). The outcome measures assessed included: ① Mental State, evaluated using the Mini-Mental State Examination (MMSE), ② Activities of Daily Living (ADL), and ③ Cognitive Function, assessed using the Alzheimer’s Disease Assessment Scale-Cognitive Subscale (ADAS-cog).

### 3.2 Assessment of literature quality and evaluation of Bias risk

The quality assessment focused on six specific domains: random sequence generation, deviations from expected interventions, incomplete outcome data, selective reporting of results, blinding of participants and outcome assessors, and overall study quality. Random sequence generation was assessed to determine whether the studies properly randomized participants, a crucial factor in minimizing selection bias. The risk of bias for random sequence generation was unclear in 19 studies, indicating incomplete reporting of randomization procedures. The remaining studies exhibited a low risk of bias, suggesting adherence to rigorous randomization protocols.

Regarding deviations from expected interventions, 22 studies exhibited unclear risk, meaning their adherence to the study protocol was not fully reported, while 3 studies demonstrated high risk, indicating significant protocol deviations. The remaining studies showed low risk in this domain. Incomplete outcome data, often a source of attrition bias, was judged to be unclear in 15 studies due to incomplete reporting on participant follow-up. However, the majority of studies adequately reported outcome data, minimizing the risk of bias.

**Figure 2:**
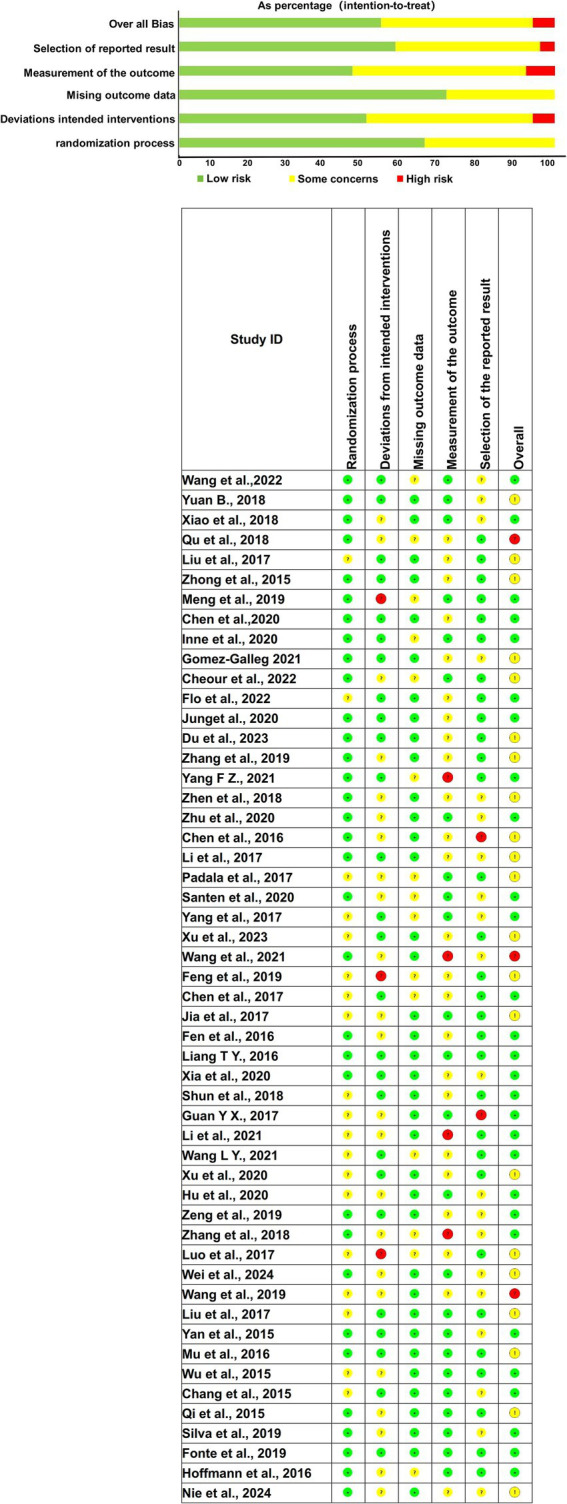
Quality assessment diagram and risk of bias summary. (Green, yellow, and red indicate low, moderate, and high risk levels, respectively. Columns represent: Randomization process, Deviations from intended interventions, Missing outcome data, Measurement of the outcome, Selection of the reported result, and Overall Bias).

Selective reporting, which occurs when only favorable outcomes are reported, was unclear in 20 studies and high risk in 2 studies, suggesting potential bias in outcome reporting. Blinding was largely adequate across most studies, reducing detection and performance bias. Overall, the comprehensive risk of bias assessment revealed that 3 studies had a high risk of bias, 28 studies were classified as low risk, and 21 studies exhibited an unclear risk of bias. The evaluation of bias risk is further detailed in Figure 2, which illustrates the distribution of potential biases across all included studies. Despite some concerns regarding the reporting quality, the overall methodological rigor of the included studies supports the validity of the findings.

### 3.3 Results of network meta-analysis

#### 3.3.1 Network structure diagram

**Figure 3:**
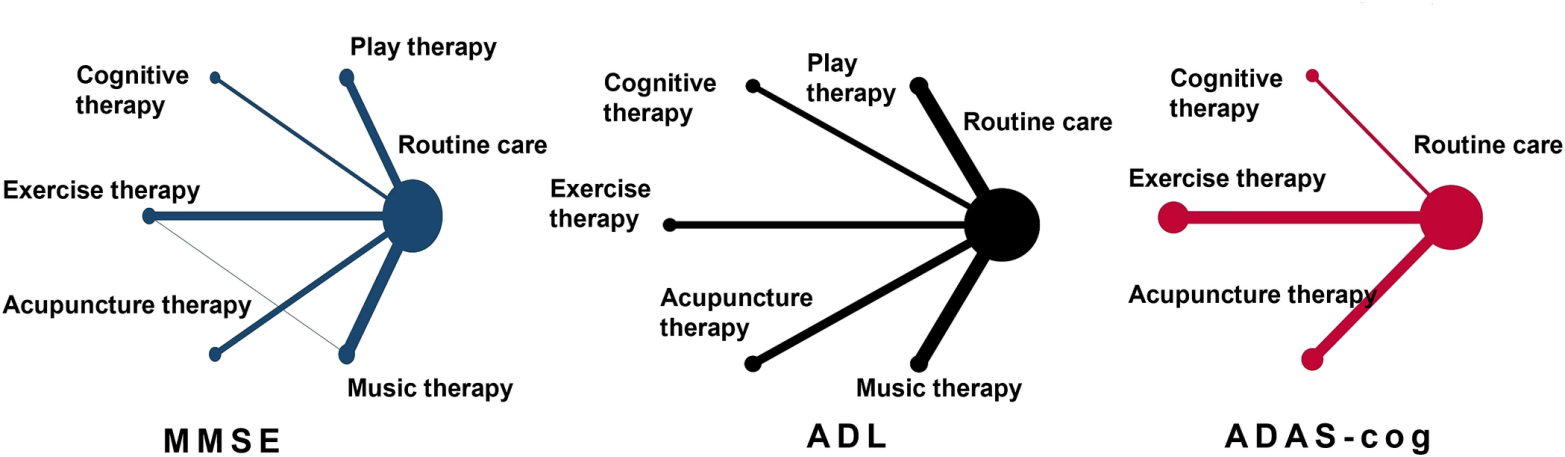
Evidence network diagram of network meta-analysis comparisons. The size of the nodes represents the sample size. The thickness of the lines represents the number of studies included in the comparison.

As depicted in Figure 3, the network diagram provides a visual representation of direct and indirect comparisons across the different physical therapy interventions. Direct comparisons were particularly robust between the control group (routine care) and various therapies, notably play therapy, exercise therapy, acupuncture therapy, and cognitive therapy. However, direct head-to-head comparisons between these specific therapies were less frequent, with limited studies available for pairwise comparisons between music therapy and other interventions. This underlines the importance of integrating indirect evidence through network meta-analysis to establish a more complete comparative efficacy profile across all interventions.

#### 3.3.2 Heterogeneity test

The heterogeneity across studies was evaluated using the inconsistency factor for each outcome (MMSE, ADL, ADAS-cog). Inconsistency factors ranged from 0 to 5.305, with 95% confidence intervals including 0. This suggests a high level of consistency between direct and indirect comparisons, indicating minimal heterogeneity and supporting the validity of the network model. The absence of significant inconsistency strengthens the reliability of the findings and suggests that the interventions have been evaluated in a comparable manner across different trials, allowing for meaningful comparisons.

#### 3.3.3 MMSE outcome

The MMSE outcome was assessed across five physical therapy interventions: music therapy, acupuncture therapy, play therapy, cognitive therapy, and exercise therapy. The control group received standard care, routine nursing, or daily activities. Play therapy showed the most significant improvement in MMSE scores (MD = 0.03, 95% CI: 0.01–0.22, *p* < 0.05), reflecting its ability to engage patients cognitively in an interactive manner that may stimulate neuroplasticity. Cognitive therapy also exhibited strong efficacy (MD = 0.14, 95% CI: 0.02–1.29, *p* < 0.05), likely due to its focus on targeting specific cognitive deficits such as memory and executive function through structured tasks. Exercise therapy (MD = 0.13, 95% CI: 0.02–0.85, *p* < 0.05), acupuncture therapy (MD = 0.13, 95% CI: 0.02–1.06, *p* < 0.05), and music therapy (MD = 0.04, 95% CI: 0.01–0.20, *p* < 0.05) were also effective in enhancing mental state, but to a lesser extent compared to play and cognitive therapies. These findings suggest that interventions focusing on both physical and cognitive engagement may offer the greatest benefits in improving cognitive outcomes in AD patients.

#### 3.3.4 ADL outcome

The ADL outcome, which measures functional independence, showed that all five physical therapies—music therapy, acupuncture therapy, play therapy, cognitive therapy, and exercise therapy—were effective compared to the control group. Play therapy (MD = 0.01, 95% CI: 0.03–0.05, *p* < 0.05) emerged as the most effective, followed by cognitive therapy (MD = 1.19, 95% CI: 0.16–0.32, *p* < 0.05) and exercise therapy (MD = 0.84, 95% CI: 0.01–0.16, *p* < 0.05). Play therapy’s leading efficacy may be attributed to its capacity to combine physical and cognitive tasks, encouraging motor function and cognitive interaction simultaneously. Exercise therapy’s improvements in ADL reflect its ability to enhance physical strength and balance, important for maintaining independence in AD patients.

#### 3.3.5 ADAS-cog outcome

For cognitive function measured by the ADAS-cog, acupuncture therapy showed the strongest improvement (MD = 0.02, 95% CI: 0.03–0.36, *p* < 0.05), followed by cognitive therapy (MD = 0.03, 95% CI: 0.01–0.60, *p* < 0.05), and exercise therapy (MD = 0.06, 95% CI: 0.01–0.48, *p* < 0.05). The pronounced effect of acupuncture on cognitive improvement is consistent with its proposed neuroprotective mechanisms, including modulation of neurotransmitter levels and reduction of amyloid-beta accumulation. This suggests that acupuncture may be particularly effective for patients in earlier stages of cognitive decline. Music therapy (MD = 0.94, 95% CI: 0.32–2.73, *p* < 0.05) also demonstrated a notable effect, potentially due to its role in improving mood and reducing anxiety, which can positively affect cognitive outcomes.

### 3.4 Ranking of network meta-analysis results

#### 3.4.1 MMSE outcome

The probabilistic ranking of the five interventions for MMSE improvement was play therapy > music therapy > acupuncture therapy > exercise therapy > cognitive therapy > routine nursing (Table 3). Play therapy’s leading rank underscores its comprehensive benefit in cognitive stimulation, potentially due to its ability to engage multiple cognitive domains simultaneously, such as problem-solving, memory, and attention.

**Table 3:** Probability ranking results of various physiotherapy interventions for patients with AD (%).

Intervening measure	MMSE	ADL	ADAS-cog
Standard Therapy	1.7	3.5	0.6
Play Therapy	81.5	98.3	-
Cognitive Training Therapy	45.0	40.9	72.1
Exercise Therapy	46.5	52.3	54.7
Acupuncture Therapy	47.9	49.6	72.5
Music Therapy	77.3	22.6	-

#### 3.4.2 ADL outcome

For ADL improvement, the ranking was play therapy > exercise therapy > acupuncture therapy > cognitive therapy > music therapy > routine nursing (Table 3). This result aligns with the hypothesis that interventions combining cognitive and physical engagement, like play therapy, may provide the greatest functional benefits, particularly in maintaining or improving daily living activities.

#### 3.4.3 ADAS-cog outcome

The ranking for ADAS-cog was acupuncture therapy > cognitive therapy > exercise therapy > routine nursing (Table 3). The prominent role of acupuncture in improving cognitive function, as measured by ADAS-cog, suggests its potential as an adjunctive therapy for cognitive enhancement in AD patients, particularly in combination with other physical or cognitive interventions.

#### 3.4.4 Publication bias

**Figure 4:**
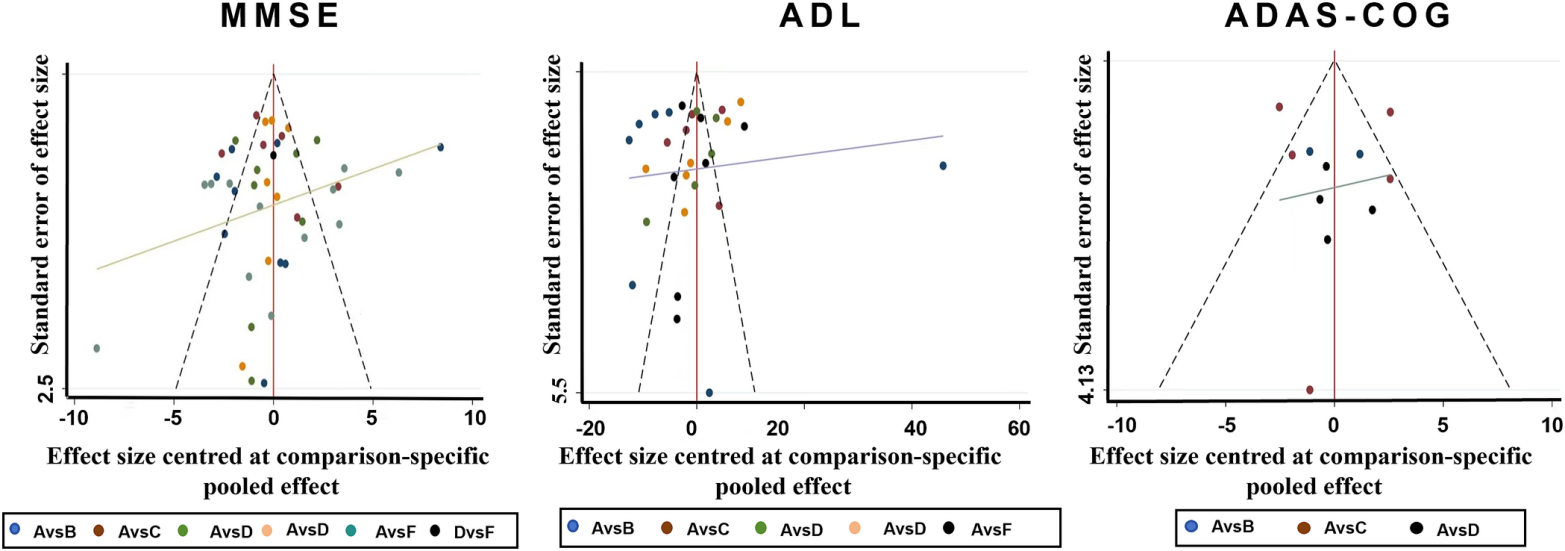
Funnel plots for various physiotherapy from all of the included studies. A. Routine care, B. Play therapy, C. Cognitive therapy, D. Exercise therapy, E. Acupuncture therapy, F. Music therapy.

The funnel plot revealed asymmetry, indicating potential publication bias across some outcomes (Figure 4). The risk of publication bias, particularly in smaller studies with significant results, highlights the need for cautious interpretation of certain findings. Future studies should aim for pre-registration and adherence to transparent reporting standards to mitigate this issue.

#### 3.4.5 Sensitivity analysis

Sensitivity analysis, performed by systematically excluding individual studies, revealed no significant changes in the effect estimates, indicating that the findings of the network meta-analysis are robust and not unduly influenced by any single study. This strengthens confidence in the consistency and reliability of the overall findings.

#### 3.4.6 Subgroup analysis

Subgroup analysis focusing on the duration of interventions suggested that interventions with shorter durations (<12 weeks) had more pronounced effects. However, due to the limited availability of data for longer-term studies, conclusions about the long-term efficacy of these interventions remain tentative, warranting further research with extended follow-up periods.

#### 3.4.7 Subgroup analysis of the impact of play therapy on MMSE

Play therapy with a duration of less than 12 weeks (*p* = 0.03) demonstrated significantly greater MMSE improvements compared to the control group. This suggests that short-term, intensive play therapy may be particularly effective in improving cognitive outcomes in AD patients.

#### 3.4.8 Subgroup analysis of the impact of play therapy on ADL

Play therapy lasting less than 12 weeks (*p* < 0.00001) also showed a significant impact on ADL improvements, reinforcing the short-term benefits of this intervention for functional outcomes in AD patients. These findings support the implementation of short-term, targeted interventions in clinical settings to yield immediate functional benefits.

#### 3.4.9 Subgroup analysis of acupuncture therapy on ADAS-cog

Acupuncture interventions of less than 12 weeks (*p* = 0.0001) led to significant improvements in ADAS-cog scores, highlighting the potential for acupuncture to offer meaningful cognitive benefits within a relatively short period. Future studies should explore the optimal duration and frequency of acupuncture for sustained cognitive improvements in AD patients (see Table 4).

**Table 4:** Subgroup analysis of optimal physical therapy interventions.

Study intervention characteristics	Number of included studies	Total number of cases	Heterogeneity test results		MD(95%CI)	Meta-analysis results	
			$I^{2}$ (%)	*p* value		Z value	*P* value
MMSE treatment cycle in play therapy (week)							
<12	3	148	0	0.57	1.06 (0.13 ~ 2.00)	2.23	0.03
≥12	6	631	97	<0.00001	4.26 (0.22 ~ 8.29)	2.07	0.04
ADL treatment cycle in play therapy(week)							
<12	3	144	84	0.002	1.76 (2.23 ~ 5.75)	0.87	0.39
≥12	4	351	100	<0.00001	20.3(−1.21 ~ 41.81)	1.85	0.06
ADAS-cog treatment cycle in acupuncture therapy(week)							
<12	2	120	0	0.9	−4.11(−6.19 ~ −2.03)	3.87	0.0001
≥12	2	173	0	0.48	−2.73(−5.55 ~ 0.10)	1.89	0.06

## 4 Discussion

Alzheimer’s disease, a chronic neurodegenerative condition, currently lacks specific medical treatments or interventions capable of reversing its progression (Huang and Wei, 2016). A comprehensive review of the pertinent literature indicates that while researchers have undertaken extensive clinical investigations, these studies largely focus on pharmacological and physical therapies, with no consensus regarding the most effective specific intervention. The inherent limitations of pharmacological treatments, including adverse effects, high costs, and limited long-term efficacy, underscore the growing need for non-pharmacological alternatives that are both effective and accessible. Physical therapies are distinguished by their simplicity, affordability, and safety, which positions them as attractive complementary options in AD management. Consequently, this study utilizes a network meta-analysis to evaluate the efficacy of five distinct physical therapies for patients with AD and employs the Surface Under the Cumulative Ranking (SUCRA) method for ranking. The objective is to offer more reliable, efficient, and targeted intervention options for individuals with AD. Through this method, we were able to integrate both direct and indirect comparisons across multiple interventions, thus providing a comprehensive ranking that reflects real-world clinical outcomes. The network meta-analysis allowed us to compare and rank multiple interventions simultaneously, providing a robust framework for determining the most effective therapeutic approaches. The results of cluster stratification suggest that play therapy and acupuncture therapy constitute the most effective interventions for enhancing the conditions of patients with AD.

Further subgroup analysis indicates that play therapy administered for a duration of less than 12 weeks yields a more pronounced improvement in MMSE and ADL scores among AD patients. Conversely, acupuncture therapy of the same duration demonstrates a more substantial impact on the ADAS-cog scores. These findings emphasize the importance of aligning specific interventions with the targeted outcomes of AD patients, as cognitive, functional, and emotional improvements may require different therapeutic approaches.

Based on assessments of MMSE and ADL scores, play therapy emerged as the most effective intervention. As an innovative therapeutic approach, play therapy presents several advantages, including high entertainment value, flexibility in forms, ease of patient acceptance, cost-effectiveness, and its potential to improve family relationships. Group play activities, in particular, offer cognitive and social stimulation, helping to alleviate the social isolation commonly experienced by AD patients. Studies have shown that participation in games can enhance limb function, stimulate mental engagement, and reduce sedentary behavior in patients with AD. Group game activities provide more opportunities for social interaction, increasing communication and alleviating loneliness, effectively enhancing subjective well-being and, consequently, improving mental state and daily living abilities in AD patients. Further investigation into the physiological mechanisms reveals that play therapy can stimulate cognitive activity, expand thinking, continuously stimulate brain cells, slow down brain degeneration, accelerate cerebral blood flow, facilitate neuroplasticity and functional reorganization, promote glucose metabolism in the anterior cingulate cortex, and fully engage positive emotions, improving cognitive function and physical coordination, (Chen, 2012; Schroder et al., 2007) thereby positively influencing the mental state and daily living abilities of AD patients. Music therapy showed a secondary effect on MMSE scores. As an emerging interdisciplinary field, music therapy, based on psychological treatment theories and methods, utilizes specially designed musical activities and experiences to exert the unique physiological and psychological effects of music, aiming to eliminate psychological barriers and promote rehabilitation (Pongan et al., 2017). The effectiveness of music therapy in improving patient mood and alleviating heart and respiratory rates has been confirmed. It is widely used in settings such as operating rooms and outpatient infusion centers. Some scholars have also demonstrated that music therapy can significantly improve cognitive function and quality of life in AD patients (Palisson et al., 2015). Exercise therapy showed a secondary effect on ADL scores. Investigation into the physiological mechanisms reveals that exercise can promote blood flow to the brain, accelerate angiogenesis, enrich the brain’s microvascular network, and trigger neuroplasticity in brain regions (Lista and Sorrentino, 2010). Exercise can promote the expression of brain-derived neurotrophic factor and vascular endothelial growth factor, promoting neuronal morphological changes and enhanced synaptic plasticity, thereby improving brain structures and neural circuits involved in cognition (Pereira et al., 2007). Exercise can also inhibit excessive astrocyte activation, slowing down the abnormal accumulation of Aβ and Tau proteins, thereby improving cognitive and memory function (Zhang X. L. et al., 2019; Lu et al., 2017).

Acupuncture therapy has also been shown to positively influence the mental state and daily living abilities of patients with AD. When evaluating interventions aimed at enhancing cognitive function scores in AD patients, acupuncture therapy emerges as the most effective option. Acupuncture, grounded in traditional Chinese medicine, offers a holistic approach to managing AD by targeting underlying imbalances in the body’s energy pathways, known as meridians. Recent advances in neuroimaging techniques have also provided evidence that acupuncture can modulate specific brain networks associated with cognitive processing and emotional regulation. This further supports the therapeutic value of acupuncture beyond its traditional framework. In recent years, the progressive development of traditional Chinese medicine has garnered significant interest from researchers in the field of acupuncture. According to traditional Chinese medicine, the pathology of Alzheimer’s disease is localized in the brain, and the Governing Vessel is intimately connected to cerebral functions. In accordance with the principles of syndrome differentiation and treatment in traditional Chinese medicine, external therapeutic modalities such as electroacupuncture, meridian acupuncture, and head acupoint stimulation have demonstrated specific therapeutic efficacy in enhancing cognitive functions and decelerating disease progression in patients with AD (Guan, 2018). A thorough review of extensive literature reveals that head acupoints, including Baihui, Sishencong, Zusanli, and Xuan Zhong, are frequently selected in acupuncture treatments. The Baihui acupoint, situated at the juncture of the midline of the head and the line connecting the two ears, is intimately associated with cerebral functions. It plays a pivotal role in regulating brain activity, balancing yin and yang, and is purported to enhance cognitive alertness and sensory perception. Acupuncture at the Baihui point is believed to nourish the kidneys and marrow, thereby contributing to the improvement of brain function. The Xuan Zhong acupoint, often referred to as the “marrow meeting” point, is instrumental in nourishing the kidneys and essence, as well as replenishing the marrow. The selection of the Zusanli acupoint is effective in regulating the spleen and stomach, in addition to augmenting qi and blood. The Sishencong acupoint is noted for its calming properties (Song et al., 2019). Acupuncture treatment can facilitate the promotion of qi and blood circulation, regulation of the spirit, and enhancement of cognitive functions. Consequently, it holds potential for improving cognitive abilities and delaying the aging process, thereby exerting a beneficial impact on the cognitive function of patients with AD.

Moreover, although the precise mechanisms by which acupuncture exerts therapeutic effects on cognitive function in patients with AD are not yet fully elucidated, existing literature, particularly studies conducted on animal models (Cao et al., 2014; Jang et al., 2023), indicates several plausible pathways. Acupuncture may improve cognitive function by decreasing the levels of amyloid-beta (Aβ) protein in the brains of mice exhibiting dementia. Additionally, it appears to reduce the deposition of Aβ protein within vascular structures and to enhance the synthesis and release of acetylcholine, a critical neurotransmitter implicated in learning and memory processes. Furthermore, acupuncture has the potential to enhance the functionality of hippocampal neurons, a region integral to memory formation, and to augment the antioxidant capacity of brain tissue. These mechanisms align with the pathology of AD, wherein Aβ deposition and cholinergic deficits are major contributors to cognitive decline, thus providing a strong theoretical basis for acupuncture’s role in AD management. These findings provide theoretical insights into the mechanisms through which acupuncture may ameliorate memory and cognitive functions in patients with AD. Cognitive therapy demonstrated a secondary effect on ADAS-cog scores. Further investigation into the underlying physiological mechanisms reveals that cognitive function, particularly attention, is often impaired due to brain tissue damage and atrophy (Martinez-moreno et al., 2016). Researchers should fully leverage the plasticity of the human nervous system to implement early functional rehabilitation of the damaged nervous system. The efficacy of cognitive training for AD stems from the frequent activation of the ascending reticular activating system and the limbic system by language and behavioral stimuli (Yang et al., 2015).

However, this study has several limitations. First, the study included only published articles and journals, which may introduce potential bias or geographic limitations due to variations in patient demographics, including gender, age, region, and country. Second, some included journals had small sample sizes, potentially reducing statistical power and increasing the uncertainty of the results. This increases the risk of selection bias and reduces the reliability and interpretability of the findings. Future research requires more high-quality studies with larger sample sizes. Third, some included studies may not have implemented blinding or allocation concealment, or may not have excluded non-randomized studies, potentially introducing bias into the results. Fourth, the study included participants who were able to participate in randomized controlled trials, limiting the generalizability to older adults with limited mobility or those who are very elderly. Fifth, the model consistency checks may not have fully considered potential differences between direct and indirect evidence, potentially affecting the interpretation of the results and the credibility of the model. Finally, the analysis focused on different types of physical therapy, but the included studies lacked consistent data on the duration and frequency of different physical interventions, hindering in-depth analysis. Future research could benefit from employing more standardized data collection methods to facilitate comprehensive subgroup analyses aimed at determining optimal intervention duration.

In summary, play therapy administered for a duration of less than 12 weeks has been identified as the most effective intervention for enhancing the mental state and daily living abilities of patients with AD. Conversely, acupuncture therapy, when applied for a period of less than 12 weeks, emerges as the optimal intervention for improving cognitive function in AD patients. When formulating intervention plans, it is imperative to comprehensively consider not only the intervention period and frequency but also factors such as the type, duration, and intensity of the physical therapy, as well as the age and gender of the patients. Future personalized intervention strategies should also incorporate patient preferences, caregiver involvement, and social support structures to ensure optimal adherence and therapeutic success. This holistic, individualized approach will ensure that the physical interventions are tailored to the specific needs of AD patients, thereby optimizing treatment outcomes and enhancing their quality of life. This holistic approach will yield more reliable recommendations for the prevention and treatment of clinical Alzheimer’s Disease.

## 5 Conclusion

This network meta-analysis highlights the differential therapeutic effects of various non-pharmacological interventions on AD. Game therapy and acupuncture therapy emerged as the most effective treatments for improving cognitive function, mental state, and daily living abilities in AD patients. Specifically, game therapy demonstrated significant short-term benefits for mental health and functional abilities, while acupuncture therapy showed superior long-term improvements in cognitive function. These findings underscore the importance of tailored interventions that address both cognitive and physical aspects of AD. Moving forward, incorporating such personalized therapies into clinical practice may enhance treatment outcomes and improve the quality of life for AD patients. Future research should focus on further exploring the mechanisms underlying these interventions and optimizing their application for diverse AD populations.

## Data Availability

The raw data supporting the conclusions of this article will be made available by the authors, without undue reservation.
